# Heart-related mortality after postoperative breast irradiation in patients with ductal carcinoma in situ in the contemporary radiotherapy era

**DOI:** 10.1038/s41598-021-82263-8

**Published:** 2021-02-02

**Authors:** Yu Jin Lim, Jaemoon Koh

**Affiliations:** 1 Department of Radiation Oncology, Kyung Hee University Medical Center, Kyung Hee University College of Medicine, 23 Kyungheedae-ro, Dongdaemoon-gu, Seoul, 02447 South Korea; 2 Department of Pathology, Seoul National University Hospital, Seoul National University College of Medicine, Seoul, South Korea

**Keywords:** Epidemiology, Medical research, Cancer, Breast cancer

## Abstract

Although radiation-induced cardiotoxicity has been addressed, its prognostic relevance to modern radiotherapy (RT) techniques is unclear. This study assessed the impact of adjuvant RT on heart-related deaths in patients with ductal carcinoma in situ. Patients who underwent adjuvant RT after breast-conserving surgery between 1988 and 2008 were identified from the Surveillance, Epidemiology, and End Results database. Kaplan‒Meier and competing risks analyses were conducted after propensity score-matching according to tumor laterality. A total of 41,526 propensity-matched patients were identified (n = 20,763 for either left- or right-sided tumor). In the analysis of the cumulative incidence of heart-related mortality events, there was a greater risk increment in the left-sided group over the first to second decades after RT in patients aged ≤ 50 years (*P* = 0.048). Competing risks analysis of the young patients showed that left-sided RT was associated with higher heart-related mortality rates (Grey’s test, *P* = 0.049). The statistical significance remained after adjusting for other covariates (subdistribution hazard ratio 2.35; 95% confidence interval 1.09‒5.10). Regarding the intrinsic effect of modern RT techniques, further strategies to reduce heart-related risks are needed for young patients. Close surveillance within an earlier follow-up period should be considered for these patients in clinics.

## Introduction

Postoperative irradiation of breast malignancy inevitably results in incidental radiation exposure to the heart. Over the past few decades, the cardiotoxicity following the ipsilateral breast radiotherapy (RT) has been discussed^1–3^. However, it is still unclear on whether modern breast RT techniques still induce clinically significant cardiac-specific risks. The temporal pattern of the cardiac events has yet to be discovered.

To evaluate the hazardous effects, the need for a longer follow-up duration for at least 10‒20 years has been considered a major obstacle for prospective randomization. Since the latent period of the phenomena is sometimes longer than the time interval to cancer relapse, the prognostic implications of RT-related cardiac toxicity can be underestimated. Additionally, the confounding effects of chemotherapy make the detection of RT-related cardiac dysfunction difficult in clinics. Regarding the cardiotoxic effects of major chemotherapeutic agents in breast cancer^4,5^, heart-related risks originating solely from the radiation in diverse clinical settings are not easily evaluated. Therefore, previous large-scale studies could not reach the uniform consensus on the heart-specific morbidities following breast RT^6–9^.

Here, we evaluated the cardiac mortality inherently induced by postoperative adjuvant RT in patients with ductal carcinoma in situ (DCIS) who underwent left- or right-sided breast irradiation following breast-conserving surgery. Competing risks analysis methods were used to assess the individual susceptibility to heart-related death. The present results provide useful insights into the prognostic implications for post-RT cardiotoxicity and the need for surveillance strategies in the contemporary treatment era.

## Results

### Patient characteristics

Based on the eligibility criteria, 42,528 patients were initially identified (Table 1). Left- and right-sided tumor laterality was reported in 21,765 (51%) and 20,763 (49%) patients, respectively. The proportions of patients aged ≤ 50, 51‒70, and > 70 years were 30%, 54%, and 16%, respectively. Approximately 81% of patients were Caucasian and 64% were married. Diagnosis between 2004 and 2008 was most common (47%), followed by 1999‒2003 (35%), 1994‒1998 (12%), and 1988‒1993 (5%). Patients diagnosed in the pacific coast and east regions were more common (82%) than in the other areas (18%). After propensity score-matching according to left- and right-sided tumors, the standardized difference values for the baseline covariates were reduced and acceptable (Supplementary Table 1).

**Table 1:** Baseline clinical characteristics of the study population (N = 42,528).

Variables	Number of patients (%)
**Laterality**	
Left	21,765 (51)
Right	20,763 (49)
**Age (years)**	
≤ 50	12,794 (30)
51–70	22,951 (54)
> 70	6783 (16)
**Race**	
Caucasian	34,295 (81)
African–American	3950 (9)
Others	4129 (10)
Unknown	154 (0)
**Marital status**	
Married	27,246 (64)
Not married	14,048 (33)
Unknown	1234 (3)
**Year of diagnosis**	
1988–1993	2139 (5)
1994–1998	5279 (12)
1999–2003	14,961 (35)
2004–2008	20,149 (47)
**Geographic region**	
Pacific coast	20,037 (47)
East	15,007 (35)
Northern plains	6149 (15)
Southwest	1305 (3)
Alaska	30 (0)

### Cumulative incidence rates of heart-related mortality

**Figure 1:**
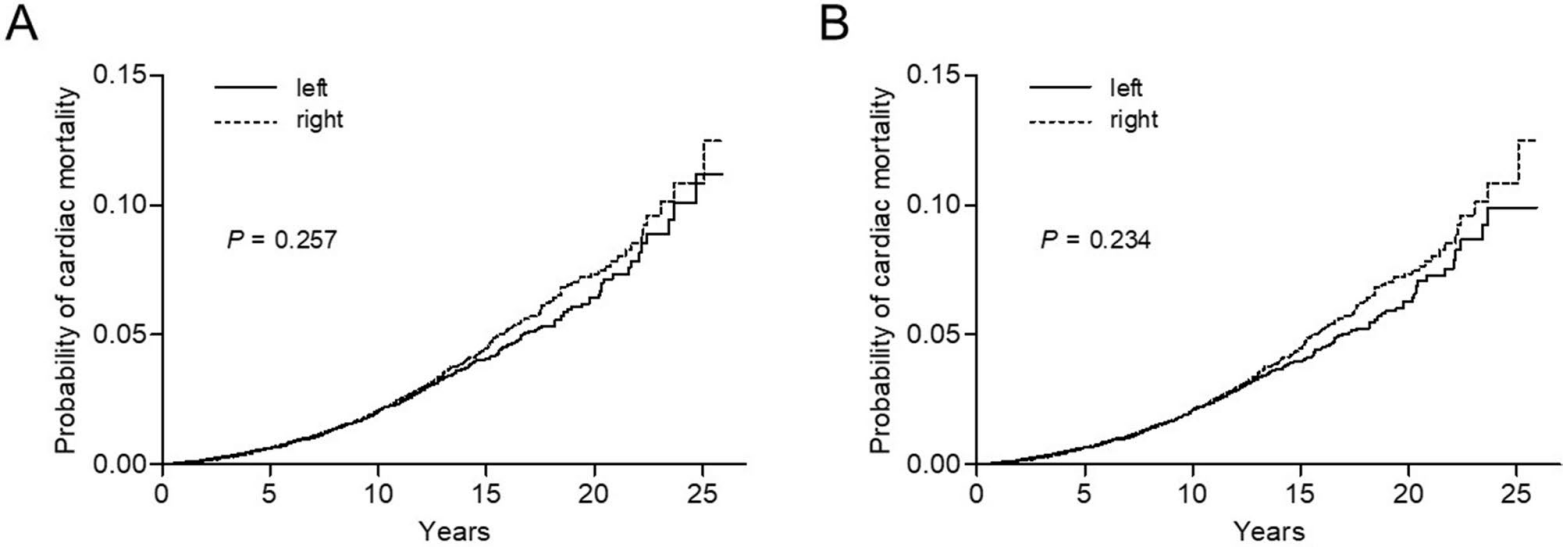
Cumulative incidence of heart-related mortality in the study population overall: (**A**) before and (**B**) after propensity score matching.

**Figure 2:**
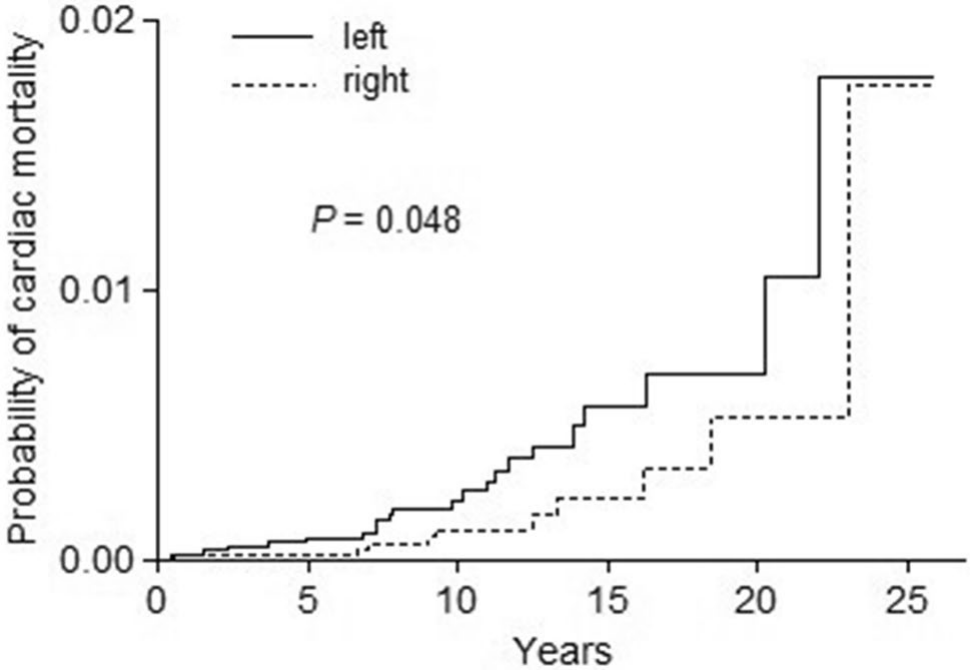
Cumulative incidence of heart-related mortality in young patients aged ≤ 50 years.

The median follow-up duration of the overall study population was 9.5 years (9.9, 9.6, and 8.6 for patients aged ≤ 50, 51‒70, and > 70 years, respectively). Figure 1 shows the cumulative incidence of heart-related death in the study population overall. Before propensity score matching, the 10-year and 20-year cardiac mortality rates of left- vs. right-sided RT groups were 2.0% vs. 2.0% and 6.4% vs. 7.3%, respectively (*P* = 0.257). In the propensity-adjusted study population, the 10-year and 20-year rates of heart-related death events in the left- vs. right-sided groups were 2.1% vs. 2.0% and 6.3% vs. 7.3%, respectively (*P* = 0.234). Overall, there were 1024, 709, and 3330 death events from heart disease, breast malignancy, and other causes (Supplementary Table 2). Stratifying patients into different age groups revealed that left-sided RT was associated with higher cardiac mortality in patients aged ≤ 50 years (*P* = 0.048, Fig. 2), whereas there were no differences in the 51‒70 and > 70-year age groups (*P* = 0.079 and 0.458, respectively). In young patients, a risk surge after 20 years of follow-up was commonly observed both in left- and right-sided disease. However, within an earlier period of the first to second decades, the risk increase was persistently greater for left-sided RT.

### Competing risks analysis by age and year of diagnosis

Table 2 lists the results of competing risks analysis of the 20-year rates for different causes of mortality. After stratifying patients according to age ≤ 50, 51‒70, and > 70 years, the long-term rates of heart-related, disease-specific, and other causes of deaths were calculated. In the univariate analysis given the competing risks, the cardiac mortality rate of left-sided tumor laterality was significantly higher than that of right-sided tumor laterality in patients aged ≤ 50 years, with estimated 20-year rates of 0.67% (95% confidence interval [CI], 0.38‒1.13) and 0.51% (95% CI 0.19‒1.15), respectively (*P* = 0.049). However, there were no significant differences between the left- and right-sided tumor for 51‒70 years (4.06% [95% CI 3.20‒5.05] vs. 5.96% [95% CI 4.80‒7.28], *P* = 0.080) and > 70 years (20.38% [95% CI 17.11‒23.85] vs. 20.59% [95% CI 17.71‒23.63], *P* = 0.574).

**Table 2:** Competing risk analysis of causes of death according to age at diagnosis.

Types of events	20-year mortality rates (%) [95% CI] by age groups								
	≤ 50 years (n = 12,557)		*P*	51–70 years (n = 22,380)		*P*	> 70 years (n = 6,589)		*P*
	Left	Right		Left	Right		Left	Right	
Heart diseases	0.67 [0.38–1.13]	0.51 [0.19–1.15]	0.049	4.06 [3.20–5.05]	5.96 [4.80–7.28]	0.080	20.38 [17.11–23.85]	20.59 [17.71–23.63]	0.574
Disease-specific	2.97 [2.16–3.98]	3.29 [2.29–4.57]	0.092	3.89 [3.14–4.76]	4.25 [3.35–5.30]	0.671	6.11 [4.50–8.05]	5.06 [3.97–6.34]	0.412
Other causes	5.09 [3.87–6.55]	3.89 [2.90–5.08]	0.910	22.25 [19.98–24.60]	18.28 [16.39–20.25]	0.194	52.24 [47.93–56.36]	53.66 [48.80–58.26]	0.636

CI: confidence interval.

The 20-year rates of heart-related deaths in patients diagnosed in 1988‒1993 were 6.04% (95% CI 4.69‒7.63) and 6.90% (95% CI 5.46‒8.56) for left- and right-sided RT, respectively, without statistical significance (*P* = 0.470). The 15-, 10-, and 5-year cardiac mortality rates of the other calendar periods (1994‒1998, 1999‒2003, and 2004‒2008) showed no significant differences between the two groups (*P* = 0.083, 0.591, and 0.402, respectively) (Supplementary Table 3).

### Competing risks regression

To evaluate the independent impact of different tumor laterality, competing risks regression analysis was conducted for the young age group. Among the patient-related variables, race, marital status, geographic region, and tumor laterality were associated with heart-related deaths in the univariate analysis (All *P* < 0.05). After adjusting for the significant covariates, left-sided tumor laterality was independently associated with higher heart-related mortality risk (subdistribution hazard ratio [sHR] 2.35, 95% CI 1.09‒5.10; *P* = 0.030) (Table 3).

**Table 3:** Competing risks regression for heart-related mortality in patients aged ≤ 50 years.

Variables	Multivariate	
	sHR (95% CI)	*P*
**Race**		
Caucasian and others	1	
African–American	3.18 (1.41–7.16)	0.005
**Marital status**		
Married	1	
Not married	1.80 (0.89–3.64)	0.100
**Region**		
Pacific coast	1	
Northern plains and southwest	1.10 (0.37–3.26)	0.860
East	2.24 (0.96–5.20)	0.062
**Laterality**		
Right	1	
Left	2.35 (1.09–5.10)	0.030

sHR: subdistribution hazard ratio; CI: confidence interval.

## Discussion

To evaluate the heart-related mortality risks of RT after breast-conserving surgery, this study analyzed the competing risks of causes of death events in patients with DCIS. Evaluation of the calendar periods indicative of modern RT techniques showed no significant difference in the study population overall. However, the cumulative incidence of patients aged ≤ 50 years showed that left-sided RT was associated with a higher incidence rate of heart-related death and also suggested the potential risk in the first to second decades following RT. Competing risks analysis revealed that left-sided RT increased the risks in the young group, even after adjusting for other related covariates. The present study proposed the prognostic implications of cardiac risks in modern breast irradiation, regardless of potential confounding effects of systemic chemotherapy.

There are several difficulties in the evaluation of the intrinsic radiation effects on heart-related deaths. First of all, the confounding effects of chemotherapy is inevitable in breast cancer treatment. Cardiac dysfunctions caused by chemotherapeutic agents such as doxorubicin, taxol, and trastuzumab have been widely discussed in breast malignancy^4,5^. Since the chemotherapy is mostly prescribed in many of patients with locally advanced breast cancer, heart-related morbidities from the sole radiation effects are not easily detected in clinics. Second, the radiation field is not uniform in advanced breast cancer. According to the extent of positive lymph nodes, the need for extended-field RT additionally covering supraclavicular lymph nodes and/or internal mammary nodal chains depends on the tumor stage, physician’s discretion, and institutional policy^10^. Regarding that the use of chemotherapy, targeted agents, or regional nodal irradiation is relatively negligible in DCIS patients, the present analysis could eliminate the related confounding effects. Comparing the outcomes of left- and right-sided tumor laterality, the long-term heart-related lethality following ipsilateral breast-only RT could be explored.

A few large-scale investigations have assessed cardiac-specific risks of adjuvant RT in patients with DCIS^8,11–13^. The landmark randomized trial initially addressed a higher heart-related mortality risk in the irradiated group, but without statistical significance (mortality rate ratio of heart disease 1.11; standard error 0.33; 2*P* > 0.1)^11^. However, a Surveillance, Epidemiology, and End Results (SEER)-based study showed a mortality ratio (vs. general population) of less than 1 (10-year standardized mortality ratio, 0.6; 95% CI 0.5‒0.7)^12^. Analysis of Swedish BCBase data showed that adjuvant RT was not associated with increased risks (hazard ratio [HR] 0.77; 95% CI 0.60‒0.98) and there was no significant difference according to tumor laterality (HR 0.85; 95% CI 0.53‒1.37 for left- vs. right-sided)^13^. Given the limited data, prior studies failed to make a uniform consensus on this topic.

The present study used analytic methods that considered other types of events as competing risks to calculate the conditional probabilities of heart-related deaths^14^. Although there were no significant differences in the study population overall, heart-related fatality was expected to be higher for left-sided RT in patients aged ≤ 50 years. When we additionally stratified the young group by calendar periods, the difference between the left- and right-sided RT was significant in 1999‒2003 but not in the earlier periods of 1988‒1993 and 1994‒1998 (data not shown). That is, the significant results in the young patients were not solely due to the relatively longer duration of follow-up. In general, elderly patients are more susceptible to miscellaneous causes of death and have higher potentials for medical comorbidities relevant to cardiovascular risks. In young patients, however, the influence of other causes of fatality or comorbid illnesses is less considerable, which might suggest a more significant prognostic relevance of post-RT cardiotoxicity. Therefore, the results of the present study suggested that young patients are an important group requiring close surveillance after breast RT.

Several large-scale studies that failed to prove the causal effect of breast RT on cardiac-specific prognosis demonstrated that advanced RT techniques might sufficiently reduce the mean heart dose^9,15^. However, dosimetric analyses revealed high doses to some regions of the heart even with contemporary RT planning^16–19^. Taylor et al. demonstrated irradiation of 20 Gy or more to heart tissue in left-sided breast RT using tangential fields^16^. Comparisons of diverse conformal planning methods showed comparable heart mean and maximum dose levels between forward-planned intensity-modulated RT and three-dimensional conformal RT (D_mean_ 2.3 ± 0.9 Gy vs. 2.6 ± 0.9 Gy; D_max_ 49.1 ± 5.0 Gy vs. 50.8 ± 3.5 Gy)^17^. Additionally, helical tomotherapy planning resulted in the highest heart V_5Gy_ (%) (26.5 ± 18.4%, *P* = 0.003). Therefore, it is unclear whether the cardiotoxic effects of more advanced RT techniques are negligible.

Analysis of cumulative incidence plots of young patients showed the highest risk surge after 20 years in both left- and right-sided breast RT, consistent with the findings of previous studies^6,8,20^. Additionally, our results showed a greater risk increment after left-sided RT, even in earlier follow-up periods of the first to second decades. The landmark study by Darby et al. demonstrated that major coronary events were initiated within 5 years of radiation exposure^21^. A recently-updated meta-analysis also showed that the risk of coronary heart disease started within 5 years and continued into the third decade of follow-up^22^. In line with the present study, we suggest that more attention is needed for potential cardiotoxic effects arising earlier, within 10 to 20-year periods after breast RT.

There are some inherent limitations of this study. Regarding the lack of data in the SEER database, previous medical history and comorbidities relevant to cardiac risks could not be considered in the present analysis. Quantitative analysis regarding RT dose or parameters was not performed in the lack of details of RT methods. Due to the small number of heart-related death events, other miscellaneous causes of mortality can mask the outcome of interest. Since cardiac tissue damage is also inducible from right-sided breast RT to some extent^18^, the study design comparing left- and right-sided RT might have underestimated the absolute effect size of post-RT cardiotoxic effects. Although the limitations weakened the meaning of our data, this study analyzed the large cohort of patients and provided population-based information that has not been well obtained from other institutional analyses.

This study evaluated the cardiac mortality risks of ipsilateral breast RT in patients with DCIS. After excluding the potential confounding effects of systemic and/or targeted chemotherapeutic agents, our results suggested that heart-related lethality of young patients needs to be considered in the modern context of RT, within the first to second decades of follow-up. The present population-based analysis provides additional knowledge of optimal radiotherapeutic and surveillance strategies regarding the inherent cardiotoxic effect of breast irradiation. Further large-scale investigations are needed to establish consensus guidelines in the era of advanced RT techniques.

## Methods

### Study population

This study used the SEER database (1973–2014), a nation-wide cancer registry in the United States. We obtained approval from the SEER through the “Research Data Agreement.”^23^ Since all individual demographic, clinical, and pathologic data were de-identified for open access, this study was not involved in identifying or storing the personal information of the included cases. Therefore, informed consent from the subjects for the present analyses was not required. All of the data extraction and analyses were conducted in accordance with relevant guidelines published by the SEER of the National Cancer Institute.

**Figure 3:**
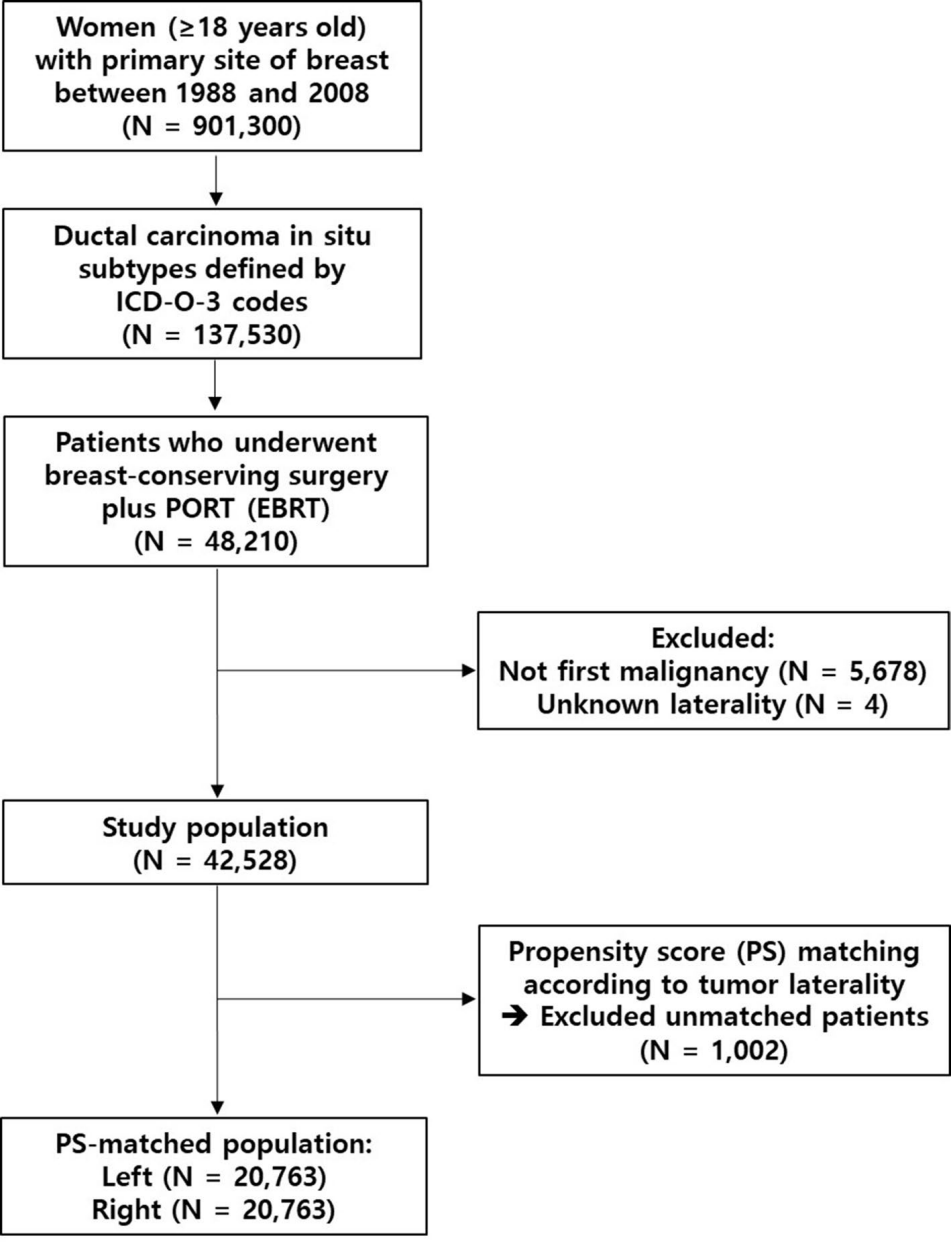
Flowchart of patient selection. PORT: postoperative radiotherapy; EBRT: external beam radiotherapy; PS: propensity score.

The SEER*Stat software (version 8.3.2; National Institutes of Health, Bethesda, MD) was used to extract raw data files of the case listing session^24^. The database contains a variety of patient- and tumor-related records, as well as survival data. The primary site of “Breast” was identified from the “Site recode ICD-O-3/WHO 2008” variable. The histologic diagnosis was confirmed based on the behavior codes of the third revision of the International Classification of Diseases for Oncology (ICD-O-3). The inclusion criteria included: (1) female, (2) age ≥ 18 years, (3) diagnosis between 1988 and 2008, (4) histology of DCIS, (5) no prior diagnosis of other malignancy, (6) unilateral tumor with complete information on breast laterality, (7) treatment with breast-conserving surgery plus postoperative RT, and (8) RT with external beam irradiation. Figure 3 shows the flowchart of the patient selection process.

### Propensity-adjusted calculation

Due to the retrospective study design, potential selection bias is a major issue affecting treatment outcomes. Given a set of baseline covariates, a propensity score is calculated as the probability of being assigned to a certain group. Our propensity-adjusted analysis compared the outcome measures between two separate groups (left- vs. right-sided breast RT). Several baseline factors were applied for the matching process, including age, race, marital status, year of diagnosis, and geographic region. A non-parsimonious logistic regression model was used to calculate propensity scores and one-to-one matching was performed based on the nearest-neighbor method without a caliper or replacement. Standardized difference values of less than 0.1 for each of the covariates were considered acceptable.

### Statistical analyses

Survival time was defined as the time interval between DCIS diagnosis and overall death. The “COD to site recode” and “SEER cause-specific death classification” variables indicated the specific reasons or organ sites that led to mortality. This study classified deaths as three types of competing events: (1) heart-related (coded as “Diseases of Heart”), (2) disease-specific [“Dead (attributable to this cancer dx)”], and (3) other causes (miscellaneous). Kaplan‒Meier analysis was used to calculate the probability of cardiac mortality and the cumulative incidence rates of the events were compared by log-rank tests. According to age and calendar periods of diagnosis, the 20-year mortality rates were estimated with competing risks analysis using Grey’s tests^25^. The prognostic association of each of the variables, such as age, race, marital status, tumor laterality, and geographic region, was evaluated in the univariate analysis. In multivariate analysis, competing risks regression with Wald tests were used to calculate the sHRs and 95% CIs^26^. *P*-values less than 0.05 were considered statistically significant. All statistical analyses were conducted using IBM SPSS Statistics 22.0 (IBM Corp., Armonk, NY, USA) and R version 3.6.0 (R Foundation for Statistical Computing, Vienna, Austria).

## Supplementary Information

Supplementary Information.
