# Supplementary material for: Heart-related mortality after postoperative breast irradiation in patients with ductal carcinoma in situ in the contemporary radiotherapy era
Source: Sci Rep. 2021 Feb 2;11:2790. doi: 10.1038/s41598-021-82263-8 (PMC7854728; doi:10.1038/s41598-021-82263-8)
Supplement: Supplementary file 1 — Supplementary Information. [file 41598_2021_82263_MOESM1_ESM.docx]

**Heart-related mortality after postoperative breast irradiation in patients with ductal carcinoma in situ in the contemporary radiotherapy era**

Yu Jin Lim^1,*^, Jaemoon Koh^2^

^1^Department of Radiation Oncology, Kyung Hee University Medical Center, Kyung Hee University School of Medicine, Seoul, South Korea

^2^Department of Pathology, Seoul National University Hospital, Seoul National University College of Medicine, Seoul, South Korea

**^*^Corresponding author:** Yu Jin Lim, M.D., Ph.D.

Department of Radiation Oncology, Kyung Hee University Medical Center, Kyung Hee University School of Medicine, 23 Kyungheedae-ro, Dongdaemoon-gu, Seoul, 02447, South Korea

*E-mail address:* [yujindw@gmail.com](mailto:yujindw@gmail.com)

**Supplementary Table 1.** Distribution of baseline variables before and after propensity score matching

| Characteristics | Before propensity score matching | | *Standardized*  *difference* | After propensity score matching | | *Standardized*  *difference* |
| --- | --- | --- | --- | --- | --- | --- |
|  | Left  (n = 21,765) | Right  (n = 20,763) |  | Left  (n = 20,763) | Right  (n = 20,763) |  |
| Age (years) |  |  |  |  |  |  |
| ≤ 50 | 6514 (30) | 6280 (30) | -0.004 | 6277 (30) | 6280 (30) | 0.003 |
| 51−70 | 11787 (54) | 11164 (54) |  | 11216 (54) | 11164 (54) |  |
| > 70 | 3464 (16) | 3319 (16) |  | 3270 (16) | 3319 (16) |  |
| Race |  |  |  |  |  |  |
| Caucasian | 17489 (80) | 16806 (81) | -0.014 | 16759 (81) | 16806 (81) | -0.005 |
| African American | 2054 (9) | 1896 (9) |  | 1915 (9) | 1896 (9) |  |
| Others | 2142 (10) | 1987 (10) |  | 2026 (10) | 1987 (10) |  |
| Unknown | 80 (0) | 74 (0) |  | 63 (0) | 74 (0) |  |
| Marital status |  |  |  |  |  |  |
| Married | 13979 (64) | 13267 (64) | 0.009 | 13247 (64) | 13267 (64) | 0.002 |
| Not married | 7173 (33) | 6875 (33) |  | 6935 (33) | 6875 (33) |  |
| Unknown | 613 (3) | 621 (3) |  | 581 (3) | 621 (3) |  |
| Year of diagnosis |  |  |  |  |  |  |
| 1988−1993 | 1094 (5) | 1045 (5) | 0.014 | 1021 (5) | 1045 (5) | -0.001 |
| 1994−1998 | 2737 (13) | 2542 (12) |  | 2535 (12) | 2542 (12) |  |
| 1999−2003 | 7714 (35) | 7247 (35) |  | 7308 (35) | 7247 (35) |  |
| 2004−2008 | 10220 (47) | 9929 (48) |  | 9899 (48) | 9929 (48) |  |
| Geographic region |  |  |  |  |  |  |
| Pacific coast | 10282 (47) | 9755 (47) | 0.005 | 9754 (47) | 9755 (47) | 0.005 |
| East | 7639 (35) | 7368 (36) |  | 7392 (36) | 7368 (36) |  |
| Northern plains | 3192 (15) | 2957 (14) |  | 2991 (14) | 2957 (14) |  |
| Southwest | 637 (3) | 668 (3) |  | 619 (3) | 668 (3) |  |
| Alaska | 15 (0) | 15 (0) |  | 7 (0) | 15 (0) |  |

**Supplementary Table 2.** Number of death events according to age and tumor laterality

|  | Number of death events (Left/Right) | | |
| --- | --- | --- | --- |
|  | Heart disease | Disease-specific | Other causes |
| Age (years) |  |  |  |
| ≤ 50 | 31 (21/10) | 142 (81/61) | 220 (111/109) |
| 51−70 | 370 (168/202) | 363 (186/177) | 1540 (795/745) |
| > 70 | 623 (305/318) | 204 (96/108) | 1570 (777/793) |
| Total | 1024 (494/530) | 709 (363/346) | 3330 (1683/1647) |

**Supplementary Table 3.** Competing risk analysis for causes of death according to calendar periods

| Types of events | Mortality rates (%) [95% CI] by year-of-diagnosis groups | | | | | | | | | | | |
| --- | --- | --- | --- | --- | --- | --- | --- | --- | --- | --- | --- | --- |
|  | 20-yr rates of 1988−1993  (N = 2,066) | | *P* | 15-yr rates of 1994−1998  (N = 5,077) | | *P* | 10-yr rates of 1999−2003  (N = 14,555) | | *P* | 5-yr rates of 2004−2008  (N = 19,828) | | *P* |
|  | Left | Right |  | Left | Right |  | Left | Right |  | Left | Right |  |
| Heart diseases | 6.04 [4.69−7.63] | 6.90 [5.46−8.56] | 0.470 | 3.78  [3.09−4.58] | 4.42  [3.66−5.27] | 0.083 | 2.28  [1.96−2.65] | 1.97  [1.67−2.31] | 0.591 | 0.42  [0.31−0.56] | 0.58  [0.45−0.75] | 0.402 |
| Disease-specific | 4.84  [3.64−6.29] | 5.25  [4.00−6.73] | 0.651 | 3.03 [2.41−3.75] | 3.26 [2.62−4.01] | 0.703 | 1.60  [1.33−1.91] | 1.40  [1.14−1.69] | 0.108 | 0.37  [0.26−0.51] | 0.36  [0.25−0.49] | 0.990 |
| Other causes | 20.35  [17.92−22.89] | 17.28  [15.04−19.66] | 0.125 | 11.97  [10.74−13.28] | 11.75  [10.52−13.04] | 0.439 | 6.92  [6.35−7.52] | 7.26  [6.68−7.88] | 0.297 | 1.94  [1.68−2.23] | 1.93  [1.67−2.22] | 0.499 |

CI: confidence interval.
